# Case Report: An Unusual Case of Pheochromocytoma

**DOI:** 10.3389/fcvm.2022.919500

**Published:** 2022-06-10

**Authors:** Ying Liao, Shanshan Shi, Lihua Liao, Yukun Zhao, Rongwen Lin, Kaihong Chen

**Affiliations:** ^1^Longyan First Affiliated Hospital of Fujian Medical University, Longyan, China; ^2^The Third Clinical Medicine College, Fujian Medical University, Fuzhou, China

**Keywords:** syncope, pheochromocytoma, long QT syndrome, malignant arrhythmias, torsade de pointes

## Abstract

Pheochromocytoma is a benign catecholamine secreting tumor, which is rare and originates from the adrenal gland. It has been known for a wide range of clinical manifestations and can mimic other difficult-to-diagnose diseases. Here, we report a female patient with acquired long QT syndrome, which is a rare complication of pheochromocytoma. Although relatively rare, the presence of pheochromocytoma should be considered in the case of malignant arrhythmias and electrocardiographic changes in patients.

## Introduction

Pheochromocytoma is a benign catecholamine-secreting tumor, which is rare and originates from the adrenal gland. It has been known for a wide range of clinical manifestations and can mimic other difficult-to-diagnose diseases. Here, we report a female patient with acquired long QT syndrome (LQTs), which is a rare complication of pheochromocytoma. Although the clinical symptoms of this case are atypical, the pathologic and computed tomography (CT) scan imaging findings of this case are typical. It is worth the communication and learning of physicians.

## Case Presentation

A 58-year-old woman came to an emergency department with a 6-day history of dizziness, headache, and syncope. Prodromal symptoms included palpitation and nausea but did not occur every time. She had no chest pain, dyspnea, fever, hematemesis, hematochezia, epileptic seizures, hemiplegia, aphasia, or urinary or fecal incontinence. Each syncope episode could be a spontaneously complete recovery, but it appeared to be unrelated to posture, emotion, or situation. She was presented to an emergency department at another institution but, unfortunately, was not diagnosed. However, her symptoms continued to worsen, and syncope episodes became more frequent (Table 1).

**Table 1:** Timeline.

July 23^rd^	The patient felt head pain, dizziness, accompanied by nausea and vomiting, and accompanied by palpitation and fatigue.
July 28^th^	Syncope occurred repeatedly for four times, with headache and palpitation before syncope.
July 29^th^	The patient experienced syncope three times.
Admission
July 29^th^	The patient experienced syncope again, ECG monitoring showed torsade de pointes (TdP) (it was a pity that the ECG was not recorded in time), which was converted with cardiopulmonary resuscitation. The patient was admitted to the coronary care unit (CCU) and started to receive further treatment.
July 31^st^	Echocardiography revealed hypertrophic ventricular wall with ejection fraction (EF) of 63%.
August 1^st^	Coronary CT angiography (CTA) suggested mild stenosis of the left anterior descending branch. Chest CT and cerebral CT were basically normal. The unenhanced abdominal CT right adrenal heterogeneous mass (45 HU) was detected.
August 2^nd^	The abdominal CT scan detected a 53 × 58 mm right adrenal heterogeneous mass.
August 14^th^	The patient underwent endoscopic retroperitoneal adrenalectomy.
September 20^th^	The patient' ECG was completely normal with QTc of 404 ms.

The patient had a medical history of hypertension. She was treated with 5 milligrams of amlodipine per day. Her blood pressure was unstable and often fluctuates, especially over the past 1 month. The patient had no history of trauma, autonomic nerve failure, epilepsy, or congenital heart disease. She did not smoke, drink, or use illegal drugs. No family history of syncope and sudden death.

Again, the patient syncope and ECG monitoring showed *torsade de pointes* (TdP) (It was a pity that the ECG was not recorded in time), which was converted with cardiopulmonary resuscitation. The patient was admitted to the coronary care unit (CCU) and further treatment was initiated. On physical examination, the temperature was 36.4°C, the respiratory rate was 20 breaths per minute, the heart rate was 74 beats per minute, the blood pressure 117/74 mmHg, and the oxygen saturation was 98%. Her cardiopulmonary and neurological examinations were normal.

**Figure 1:**
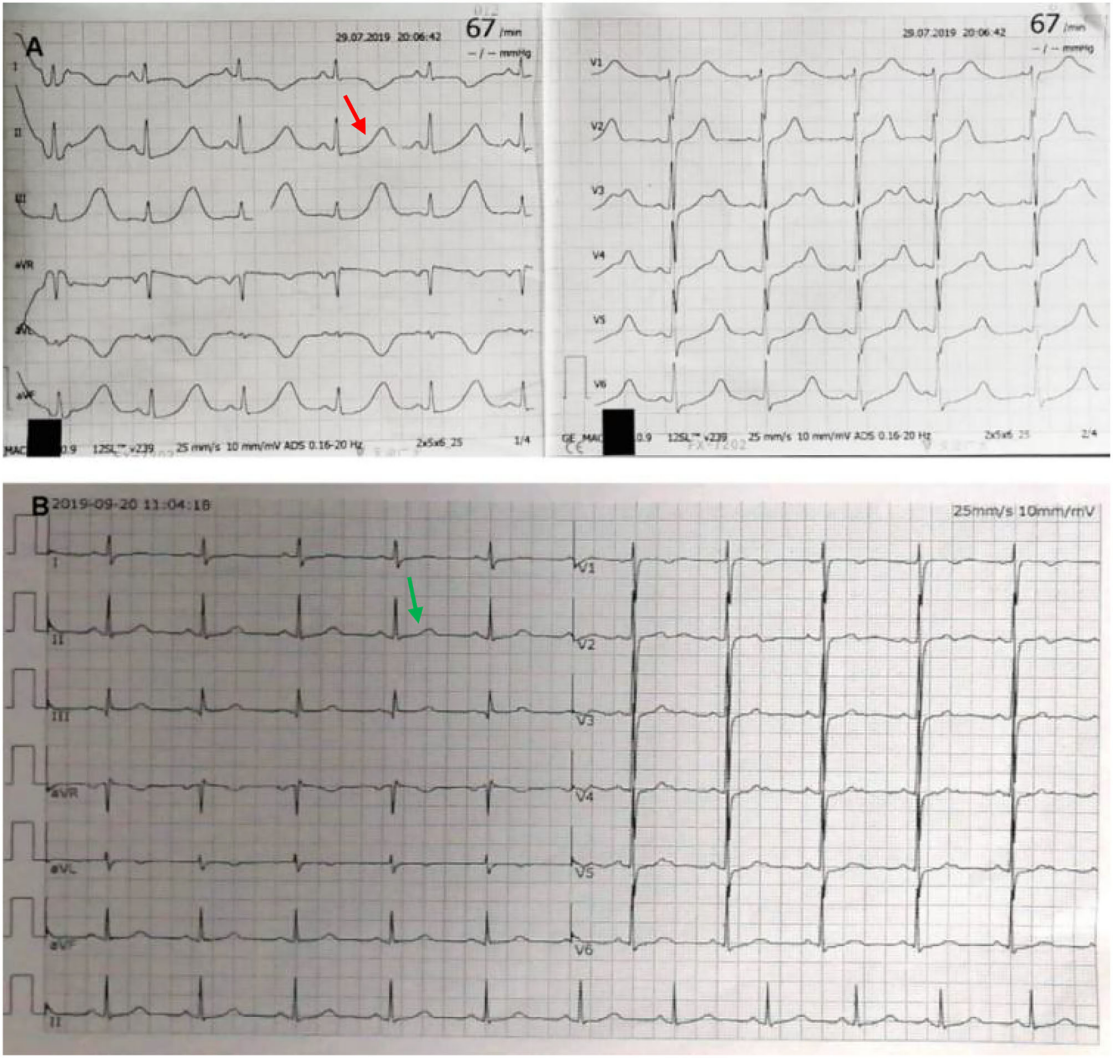
Electrocardiograph (ECG). ECG at admission shows sinus rhythm and QTc interval of 661 ms [**(A)** red arrow]. After excision of pheochromocytoma, ECG shows normalization with QTc of 404 ms [**(B)** green arrow].

Laboratory testing showed a maximum troponin I (TNI) level of 0.32 ng per milliliter (normal value, <0.3 ng per milliliter), maximum creatinine kinase-MB (CK-MB) of 41 IU per liter (normal value, <25 IU per liter), and a maximum N-terminal pro-B-type natriuretic peptide (NT-pro BNP) of 5,138.77 pg per milliliter (normal value, <450 pg per milliliter). Her fasting blood glucose was 11.46 mmol per liter (206.3 mg per deciliter) (normal value, <7 mmol per liter) [126 mg per deciliter] and hemoglobin a1c (HbA1c) of 8.3% (normal value, <6.5 %). The patient was newly diagnosed with diabetes. Hemoglobin, blood gas analysis, D dimer, serum sodium, potassium, magnesium, calcium, renal, liver, and thyroid levels were all within normal limits. The patient's electrocardiograph (ECG) showed a sinus rhythm of 67 beats per minute, ST-segment depression in V4-V6. Most notably, it showed a QTc interval of 661 ms (Figure 1A). Echocardiography revealed a hypertrophic ventricular wall with an ejection fraction (EF) of 63%. Coronary CT angiography (CTA) suggested mild stenosis of the left anterior descending branch. Chest CT and cerebral CT were normal. The patient was not taking medications that caused QT prolongation.

**Figure 2:**
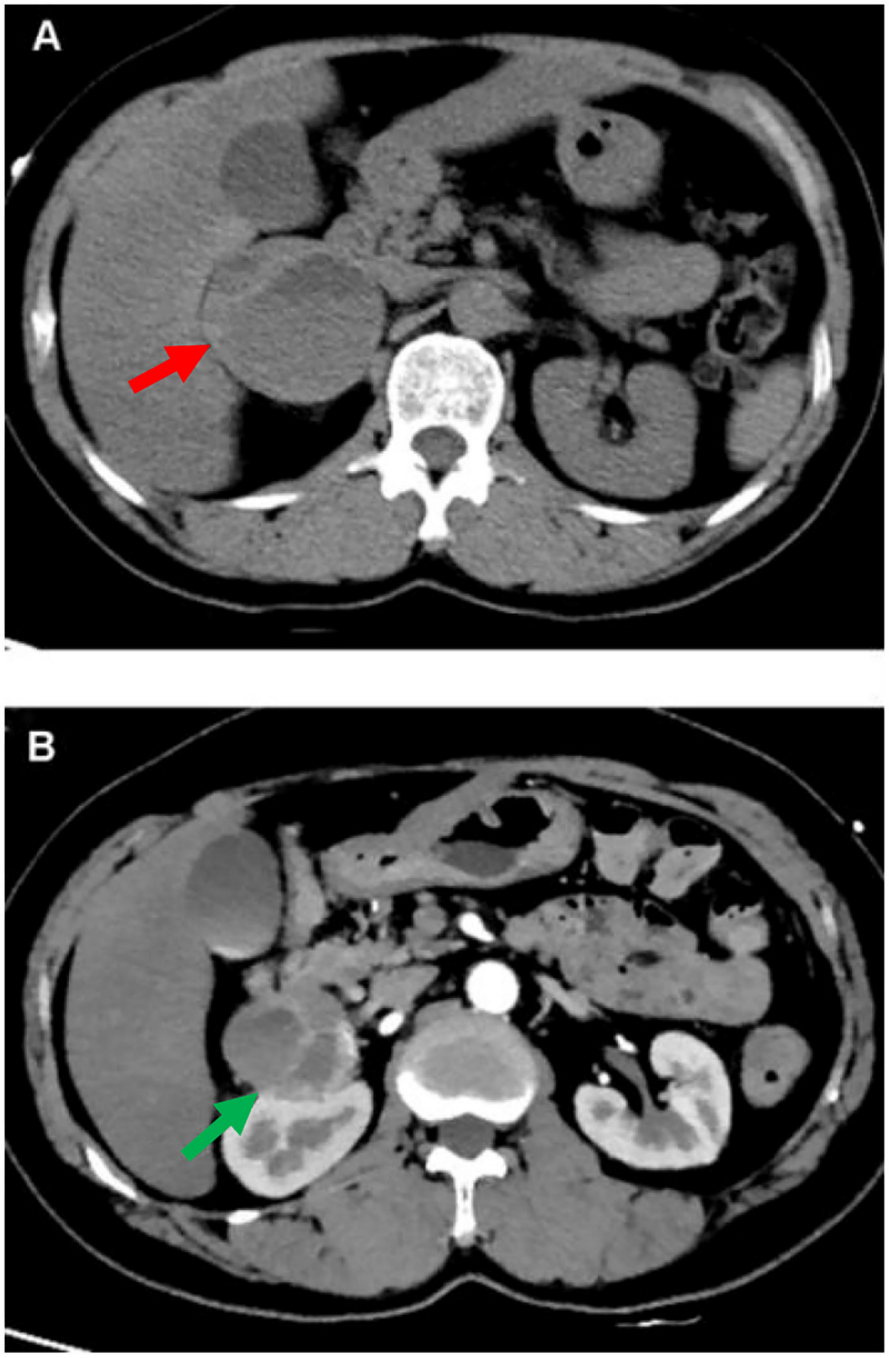
CT of abdomen. A computed tomographic (CT) of the upper abdomen (axial view) shows a 53 mm*58 mm heterogeneous tumor of the right adrenal gland [**(A)** red arrow]. Axial contrast-enhanced CT image shows the mass compose of solid enhancing component and cystic component [**(B)** green arrow].

During the hospitalization, the patient underwent an abdominal CT scan. As a result, a 53 × 58 mm right adrenal heterogeneous mass was detected. The unenhanced CT attenuation was 45 Hounsfield units (HU). Axial contrast-enhanced CT demonstrated a mass consisting of solid enhancement and cystic components (Figures 2A,B).

Adrenal endocrinology examinations were used for differential diagnosis. Plasm renin activity was 3.03 ng per milliliter per hour (normal value,1.31–3.95 ng per milliliter per hour), aldosterone was 35.38 ng per deciliter (normal value,7.6–30 pg per deciliter), morning adrenocorticotropic hormone (ACTH) was 2.84 ng per liter (normal value,10–90 ng per liter), cortisol at 8:00 568.4 nmol per liter (normal value,171–536 nmol per liter), cortisol at 16:00 457.4 nmol per liter (normal value,64–340 nmol per liter), cortisol at 0:00 450 nmol per liter (normal value,171–536 nmol per liter), 24-h urinary dopamine was 541.21 ug (normal value, <600 ug), 24-hr urinary norepinephrine was 659.63 ug (normal value, <90 ug), 24-h urinary adrenaline was 73.21 ug (normal value, <20 ug), and 24-h urinary vanillylmandelic acid (VMA) of 29.2 mg (normal value, <12 mg).

**Figure 3:**
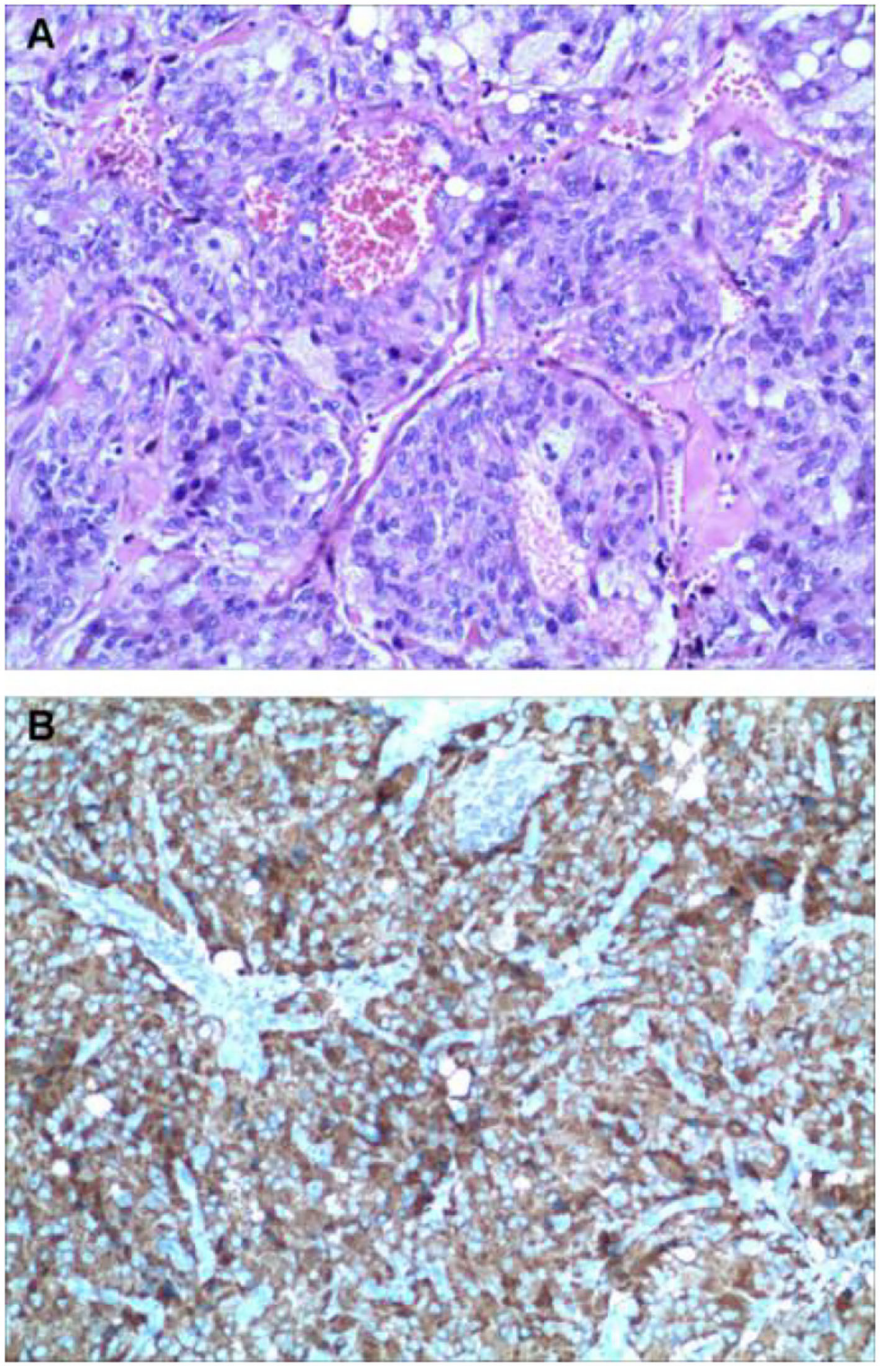
Histologic findings. Histological evaluation of adrenal neoplasms revealed rounded to polygonal cells with abundant granular amphoteric cytoplasm arranged in well-defined nests [**(A)** hematoxylin, and eosin]. Immunohistochemical testing shows positive expression of CgA **(B)**.

Two weeks later, after being prepared according to the guidelines for pheochromocytoma resection, she underwent endoscopic retroperitoneal adrenalectomy. Histopathologic examination of the adrenal mass showed that it was a typical pheochromocytoma, with large polygonal cells arranged in a thick nest and separated by a rich capillary network (zellballen appearance) (Figure 3A). Positive expression of tyrosine ki-67, CgA, Syn, and NSE on immunohistochemical examination (Figure 3B). After 1 month follow-up, the patient's ECG was completely normal with a QTc of 404 ms (Figure 1B). She is free of syncope but still has hypertension with amlodipine.

The patient does not experience syncope after surgery, and ECG indicates that QT returned to normal, confirming our assessment that pheochromocytoma is the cause of her problem.

Germline genetic testing found no mutation associated with inherited LQTs (KCNQ1, KCNH2, SCN5A, KCNE1, KCNE2, etc.), but found a mutation in SDHB associated with pheochromocytoma. SDHB mutation is a type of familial paraganglioma syndrome, which is characterized by paragangliomas with high metastatic risk in the mediastinum, thorax, abdomen, and pelvis.

Approximately 70–75% of patients with inherited LQTs have mutations, and more than one-third of patients with acquired LQTs also carry mutations in one of the major LQTs-related genes, of which KCNH2 is the more common. All the common long QT-related genes in the patient are negative. In combination with her characteristics, we considered it as an acquired LQT. Furthermore, we detect gene mutation associated with pheochromocytoma, confirming again that the etiology of the patient is pheochromocytoma.

## Discussion

Syncope is one of the most life-threatening conditions, and many people suffer from recurrent syncope without being diagnosed or treated. It is estimated that about half of people have syncope at least once in their life (1). The annual incidence of syncope in older patients was 7%, the overall prevalence was 23%, and the 2-year recurrence rate was 30%. Cardiogenic syncope is the most dangerous form, and arrhythmia is one of the most common causes of cardiogenic syncope, which is more dangerous than other etiology (2, 3).

The LQTs are an inherited or acquired heart diseases characterized by prolonged QT on the ECG, and an increased risk of life-threatening ventricular arrhythmias (4). Acquired LQTs are far more common than congenital LQTs and may be the result of pharmacologically affected electrolyte disturbances and endocrine imbalance (such as pheochromocytoma). LQTs are defined as prolongation of the QTc for heart rate, in adult males and children at >440 ms, or women at >460 ms. Approximately 16–35% of patients have varying degrees of QT prolongation, but QTc is rarely more than 600 ms as described in the literature. In our case, QTc at admission is 661 ms, which is very easy to induce TdP. TdP is a type of polymorphic ventricular tachycardia, which is characterized by a gradual change and distortion of the magnitude of the QRS complex wave around an isoelectric line on the ECG. It usually occurs in the setting of a prolonged QT interval.

Pheochromocytomas and paragangliomas (PPGLs) are rare neuroendocrine chromaffin tissue tumors that may produce catecholamines (5). About 80–85% of PPGLs come from the adrenal medulla, which is called pheochromocytoma, whereas about 15–20% of PPGLs come from the sympathetic or parasympathetic paravertebral ganglia, which is called paragangliomas (6). The prevalence of hypertension among adult outpatients ranges from 0.1 to 0.6% (7, 8). The effects of catecholamines on different organs depend on their blood concentration and the types of adrenergic receptors in the organs. So, there are highly variable clinical symptoms and signs, which can lead to delay in diagnosis, but the most common symptoms are headaches, sweating, heart palpitations, and hypertension. Serious potential cardiovascular complications include arrhythmia, hypotension, myocardial ischemia, shock, aortic dissection, cardiomyopathy, and peripheral ischemia (9). In a retrospective study of 145 subjects, arrhythmias occurred in 15 subjects. Ventricular tachycardia was found in 2 subjects, one of whom was TdP (10). Although ventricular arrhythmias are a rare complication of PPGLS, they can occur (11–16). The mechanism of QT prolongation and TdP is not very clear. We speculate that it may have been related to the presence and relative proportion of catecholamines secreted. Adrenergic stimulation can prolong the QT interval by prolonging the duration of the action potential. In addition, the increase of myocardial heterogeneity and the triggering of early afterdepolarization (EAD) are also mechanisms.

The diagnosis of pheochromocytoma is difficult owing to the rarity of the disease, the nonspecific clinical manifestations, and the uncertainty of its location. As in our case, syncope may be the first symptom, making the diagnosis of pheochromocytoma challenging and important. This case highlights the steps to diagnose pheochromocytoma. First, if pheochromocytoma is suspected, biochemical testing should be initiated, including measurement of fractionated metanephros's and catecholamines in a 24-h urine or plasma specimen. In addition, we should also be aware of false negatives and false positives in food, drugs, and other disease states, and biochemical testing should be repeated later, during episodes of symptoms that may be caused by catecholamines.

Secondly, when there is clear biochemical diagnostic evidence, the imaging study of the adrenal gland should be started. The various imaging appearances on ultrasound, CT, MRI, and functional imaging can be complementary and have characteristics that may help distinguish them from other masses of the adrenal. If MRI or CT imaging is normal and there is a strong biochemical suspicion that it is a tumor, or there is radiological evidence that it is a paraganglioma or metastatic disease, the patient should be given iodine-123 metaiodobenzyl guanidine scan and may also undergo fludeoxyglucose positron emission tomography CT (17).

Thirdly, previous studies have shown that more than 40% of patients, who develop pheochromocytoma, carry germline mutations (18, 19). Ideally, all patients with pheochromocytoma should undergo genetic testing because the results of genetic testing can better guide the clinical treatment and prognosis (20). Genetic analysis of our case reveals gene mutation in SDHB. Patients with SDHx mutations usually have head-and-neck paragangliomas, but pheochromocytomas and retroperitoneal paragangliomas are found primarily in carriers of SDHD, SDHB, and SDHA mutations (21, 22). Fortunately, no tumor was found except in the adrenal gland. After surgery, hormone levels returned to normal and symptoms disappeared, confirming the absence of tumors elsewhere. However, the long-term clinical prognosis needs to be further evaluated with follow-up.

The PPGL is pathologically characterized by a nest of tumor cells separated by surrounding capillaries, namely Zellballen. PPGLs express generic neuroendocrine markers, of which chromogranin A and synaptophysin are most often utilized, which helps distinguish them from other neuroendocrine tumors. Malignant PPGLs are defined by the presence of chromaffin cells in tissues and sites that normally do not contain these cells (23).

If pheochromocytoma is removed timely, the prognosis is favorable. However, the prognosis is poor in patients with metastases, which especially occur in patients with large and extra-adrenal tumors. After resection, patients with PPGLs have reasonable outcomes and biochemical reaction and blood pressure control are improved. All patients with PPGLs need biochemical and potentially radiological surveillance.

## Patient Perspective

“As a patient, I am glad that the cause of the disease has been found and solved. My illness came suddenly. I couldn't find the cause at first and the symptoms continued to worsen, which frightened me. I was surprised to learn that my syncope and abnormal ECG were caused by a mass in the abdominal cavity because I had never found an abdominal mass before. Finally, I am very grateful to the doctor who has treated me”.

## Conclusion

In summary, there are several important points to be learned from this case. First, when evaluating patients with common syncope symptoms, physicians need to consider both rare and severe causes. Second, pheochromocytoma can be lethal if it is undiagnosed or misdiagnosis. Thus, rapid, and correct recognition is crucial. Third, if the reversible cause of TdP is identified, surgical resection of the tumor will cure and avoid unnecessary ICD implantation.

## Data Availability Statement

The original contributions presented in the study are included in the article/supplementary material, further inquiries can be directed to the corresponding author.

## Ethics Statement

Written informed consent was obtained from the individual(s) for the publication of any potentially identifiable images or data included in this article.

## Author Contributions

All authors listed have made a substantial, direct, and intellectual contribution to the work and approved it for publication.

## Conflict of Interest

The authors declare that the research was conducted in the absence of any commercial or financial relationships that could be construed as a potential conflict of interest.

## Publisher's Note

All claims expressed in this article are solely those of the authors and do not necessarily represent those of their affiliated organizations, or those of the publisher, the editors and the reviewers. Any product that may be evaluated in this article, or claim that may be made by its manufacturer, is not guaranteed or endorsed by the publisher.
